# Enhancing Feed Efficiency and Growth in Early-Fattening Hanwoo Steers Through High-Energy Concentrate Feeding

**DOI:** 10.3390/ani15040490

**Published:** 2025-02-09

**Authors:** Emmanuel Onche, Hyunjin Cho, Andrian Deguinion Rangandang, Namkyu Kang, Suheon Kim, Hongdae Kim, Seongwon Seo

**Affiliations:** 1 Division of Animal and Dairy Sciences, Chungam National University, Daejeon 34134, Republic of Korea; oncheemmanuel1@gmail.com (E.O.); chohyunjin0927@gmail.com (H.C.); ragandangian@gmail.com (A.D.R.); 2 Sunjin Research Institute, Sunjin Ltd., Seoul 05372, Republic of Korea; nkkang@sj.co.kr (N.K.); shkim3@sj.co.kr (S.K.); hdkim@sj.co.kr (H.K.)

**Keywords:** concentrate mix, dietary energy levels, digestibility, fattening, feed efficiency, Hanwoo

## Abstract

Adequate but not excessive dietary energy supply is crucial for growth and productivity in cattle. This study aimed to evaluate the effects of three incremental changes in metabolizable energy (ME) levels in concentrate mixes, i.e., low (LEC, 10.4 MJ/kg DM), medium (MEC, 10.8 MJ/kg DM), and high (HEC, 11.0 MJ/kg DM), on growth performance and comprehensive physiological parameters in Hanwoo steers. Thirty steers, averaging 499 ± 38.0 kg, were randomly allocated to one of the treatments. Each steer received up to 8 kg of concentrate mix, with ad libitum access to tall fescue. Body weights were measured every four weeks. In week 12, rumen samples were collected, followed by fecal and blood samples. The HEC group exhibited a 16.5% higher average daily gain compared to the other groups (*p* = 0.035). Forage and total dry matter (DMI) were 46% and 12% lower in the HEC group (*p* < 0.001). Fiber digestibility increased with energy content (*p* < 0.05). Total volatile fatty acid and the acetate-to-propionate ratio decreased in the HEC group (*p* < 0.001), while blood creatinine concentration increased (*p* < 0.01). In conclusion, feeding Hanwoo steers with a high-energy concentrate mix (up to 11.0 MJ ME/kg DM) improved growth and feed efficiency without compromising rumen health.

## Simple Summary

An adequate but not excessive supply of dietary energy is crucial for growth and productivity in cattle. This 14-week feeding trial evaluated the effects of incremental changes in the energy levels in concentrate mixes on growth performance and comprehensive physiological parameters in thirty Hanwoo steers. Feeding steers with high-energy concentrates (HECs) resulted in a 16.5% higher growth rate, along with 46% and 12% lower forage and total feed intakes, respectively, compared to the other treatments. Blood creatinine concentration, a marker of muscle mass, was higher in the HEC group, as was fiber digestibility, which is associated with feed efficiency. In conclusion, feeding Hanwoo steers with a high-energy concentrate mix (up to 11.0 MJ metabolizable energy/kg dry matter) enhanced growth and feed utilization without adverse effects on health.

## 1. Introduction

Energy intake is the most critical factor influencing growth performance and farm profitability in cattle production. In Korea, as in many other countries in Asia, there is a limited supply of high-quality forage, leading to heavy reliance on lower-quality roughage sources, such as straw [1]. Consequently, feeding a high-energy concentrate mix has become a standard practice in cattle production to supply dietary energy that supports growth, maintenance, and overall productivity.

Energy is the primary nutrient required for animals. When energy intake is insufficient, even proteins are metabolized to meet energy demands [2]. Dietary energy not only supports basic maintenance and growth but also facilitates the formation of muscle and adipose tissue, both of which play a crucial role in determining meat quality and grade [3,4,5]. Insufficient energy levels can reduce growth rates, compromise immune function, and lower feed efficiency, while excessive energy intake can result in nutrient wastage, unnecessary fat accumulation, and an increased risk of metabolic disorders such as acidosis and rumen dysfunction [6,7,8]. Thus, providing adequate dietary energy during the growing and fattening periods of steers is essential to enhance production efficiency and profitability [6,9].

High-energy diets are often introduced during the fattening stage of beef cattle to promote muscle mass development and adipose tissue formation, both of which enhance carcass quality and meat grading. Studies have demonstrated that increased dietary energy levels positively affect cattle performance. For example, higher dietary energy improved weight gain, skeletal growth, and slaughter weight in steers [10]. High-energy diets also increased average daily gain (ADG) and the total fatty acid content in muscle in fattening Angus steers [11]. Additionally, previous studies reported increased dry matter intake (DMI), ADG, and serum glucose levels with higher energy intake [12,13]. Similarly, Liu et al. [14] observed increased ADG, feed efficiency, and fat accretion with higher dietary energy concentrations, along with changes in the rumen volatile fatty acid (VFA) profile toward propionic acid. Furthermore, high-energy diets can also help reduce methane (CH_4_) emissions [15,16].

In Hanwoo beef cattle, several studies have evaluated the effects of increasing energy levels in the concentrate mix to optimize growth and fattening. Kim et al. [17] reported that a high-energy concentrate mix promoted growth. They found a greater ADG and cold carcass fat content in Hanwoo steers fed concentrate mixes with a high level of total digestible nutrients (TDNs) (growing, 74%; fattening, 76% on an as-fed [AF] basis) compared with those fed low-TDN concentrate mixes (growing, 70%; fattening, 72% on an AF basis). However, other studies did not observe a positive response to feeding a higher-energy concentrate. Ahn et al. [18] indicated that four varying dietary energy levels (73.3%, 74.50%, 76.40%, and 77.10% of TDN on a dry matter [DM] basis) did not yield significant differences in growth, feed efficiency, or marbling in Hanwoo steers. Kang et al. [5] found no significant differences in growth performance when increasing the TDN levels in concentrate mixes (growing: 72.6%; early fattening: 73.1%; late fattening: 76.2%) compared with the control group (growing: 70.5%; early fattening: 71.0%; late fattening: 74%) during the growing and fattening periods. The only notable finding was that steers in the control group had a greater DMI.

Additionally, most previous studies have primarily focused on growth performance and carcass characteristics with limited emphasis on the underlying physiological and metabolic changes. Factors such as rumen fermentation profiles, nutrient digestibility, and blood metabolites play a crucial role in mediating the effects of dietary energy but remain inadequately investigated in Hanwoo cattle. A comprehensive analysis of these parameters is essential to understand the underlying mechanism better and to develop precise feeding strategies that optimize productivity while minimizing nutrient wastage and environmental impact.

Therefore, this study aimed to comprehensively evaluate the effects of incremental changes in the levels of dietary energy of concentrate mixes on intake, growth performance, nutrient digestibility, rumen characteristics, and blood metabolites. With this, we aim to provide insights into optimized feeding strategies that balance productivity and environmental sustainability.

## 2. Materials and Methods

This trial was conducted at the animal research center, Chungnam National University, South Korea. The animal use and experimental protocols were reviewed and approved by the Chungnam National University Animal Research Ethics Committee (202406A-CNU-104) before the commencement of the study.

### 2.1. Animal, Housing and Diet

Thirty 17-month-old fattening Hanwoo steers, weighing 499 ± 37.0, participated in this 14-week feeding trial study. Using a completely randomized block design, the steers were randomly allocated into three groups with blocking based on initial body weight [19]. Each group of steers was housed in a pen (10 m × 10 m) equipped with four automatic forage intake monitoring systems (Dawoon, Co., Incheon, Republic of Korea) and an automatic concentrate feeding system (Dawoon, Co., Incheon, Republic of Korea). Individual feed intake was measured automatically by the systems, identifying each animal using a radio-frequency identification tag attached to them.

Each group of steers was randomly assigned one of the three treatments with varying metabolizable energy (ME) concentrations in the concentrate mix: (1) low-energy concentrate mix (LEC, 10.4 MJ/kg DM), (2) medium-energy concentrate mix (MEC, 10.8 MJ/kg DM), and (3) high-energy concentrate mix (HEC, 11.0 MJ/kg DM). The energy levels of each concentrate mix were determined based on the Korean feeding standards for Hanwoo, where MEC represents the recommended energy level of the concentrate mix for the steer’s specific stage of growth [20]. Instead of formulating new concentrate mixes for this experiment, we selected the experimental concentrate mixes from commercial products manufactured by the Sunjin feed company based solely on their energy concentration. Unexpectedly, the bulk density of the three concentrate mixes differed significantly: MEC had the lowest bulk density (564.5 g/L), followed by HEC (618.1 g/L) and LEC (674.2 g/L) (*p* < 0.05). Detailed information on the diet formulation and chemical analysis of the treatments is provided in Table 1 and Table 2.

Each steer was fed up to 8 kg, on average, of concentrate mix daily, along with ad libitum access to tall fescue. The tall fescue was supplied twice daily at 08:00 and 18:00. The concentrate mix was automatically dispensed using an ACFS, with a set daily allowance of 8 kg. It was divided into six portions and distributed at 4 h intervals throughout the day. Clean water was provided to the steers through water cups throughout the experiment.

### 2.2. Measurement and Sample Collection

All steers had access to tall fescue and concentrate mix via the forage intake monitoring system and the automated concentrate feeding system, respectively. However, during the sampling period, the concentrate mix was fed manually by restraining the steers in stanchions to ensure that eating times were synchronized for all steers. During this period, individual concentrate intake was determined by subtracting the amount of feed refused from the amount of feed offered. Feeds used as treatments were sampled once every four weeks for chemical analysis throughout the experimental period.

Every four weeks, the recorded daily feed intakes for each steer were processed. Intakes that deviated by more than three times the standard deviation from the mean were removed as outliers. Similarly, we excluded the feed intakes on days when management operations occurred, such as bedding replacement, body weight (BW) measurement, and sampling periods. Body weight was also measured once every four weeks before the morning feeding.

At 11 weeks, feces was spot-sampled eight times over four consecutive days at nine-hour intervals (d 1: 17:00; d 2: 02:00, 11:00, 20:00; d 3: 05:00, 14:00, 23:00; and d 4: 08:00) from a total of 15 steers, with 5 steers from each treatment group. The collected fecal samples were dried at 65 °C for 72 h. The dried fecal samples from each time point were combined in equal amounts and ground together for each steer. For instance, the smallest sample weight among all time points for a given steer was used as the standard amount for pooling before grinding.

Rumen fluid was collected three times (−1, +3, and +6 h after morning feeding) over three consecutive days, following 11 weeks of study, from all steers using an oral stomach tube as previously outline by Lee et al. [21]. Initially, approximately 300 mL of rumen fluid was collected and discarded, and 400 mL was collected in a glass flask. After the collection, the pH of the rumen fluid was immediately measured, and a 10 mL sample was taken for ammonia (NH_3_-N) and volatile fatty acid (VFA) analysis. The subsamples were stored at −20 °C until analysis.

After 12 weeks, approximately 10 mL of blood was collected once from the jugular vein of each steer before the morning feeding. The blood samples were transferred into serum separator tubes (BD vacutainer; BD and CO., Franklin Lakes, NJ, USA). Blood serum was separated by centrifugation at 1300× *g* for 15 min at 4 °C and stored at −80 °C for further analysis.

### 2.3. Chemical Analyses

Chemical analyses were performed following methods described by Jeon et al. [22]. The feed and fecal matter were dried for at 60 °C for 96 h and ground through a cyclone mill (Foss, Hillerød, Denmark) fitted with a 1 mm screen. The nutrient composition of the feed samples was analyzed at Cumberland Valley Analytical Service Inc. (Hagerstown, MD, USA). The DM content (#934.15), crude protein (#990.03), ether extract (#920.39), acid detergent fiber (#973.18), and ash content (#942.05) were measured. Crude protein was estimated by multiplying the nitrogen content by 6.25, with nitrogen quantified using the Dumas method, on a Leco FP-528 Nitrogen Combustion Analyzer (Leco Inc., Saint Joseph, MI, USA). The acid detergent lignin (ADL) was measured, and neutral detergent fiber (aNDF) contents were analyzed using a heat-stable amylase, including residual ash. Additionally, soluble protein, neutral-detergent-insoluble crude protein (NDICP), and acid-detergent-insoluble crude protein (ADICP) were determined. The amount of water-soluble carbohydrate (WSC), starch, and both macro and micro minerals were also determined. For nutrient digestibility, the indigestible neutral detergent fiber (iNDF) was used as a marker. Both feed and fecal iNDF were analyzed following the protocol in Huhtanen et al. [23], with extraction occurring after incubating the samples in the rumen for 96 h. Additionally, the classification of carbohydrate and protein fractions was conducted according to Tedeschi and Fox [24] using the chemical analysis results of the feed.

The NH3-N concentration was analyzed separately for each rumen sample collected at different time points. The concentration of NH3-N in the rumen fluid was measured as follows. After recentrifuging the rumen fluid at 21,000× *g* for 15 min, 20 μL of the supernatant was mixed with 1 mL of phenol color reagent and 1 mL of alkali hypochlorite reagent. The resulting mixture was incubated in a water bath at 37 °C for 15 min. Afterward, 8 mL of distilled water was added, and the optical density of the mixture was measured at 630 nm using spectrophotometer (UV-1800, Shimadzu, Kyoto, Japan).

The VFA concentration was analyzed separately for each rumen sample collected at different time points. To measure the VFA concentration, 1 mL of rumen fluid supernatant was mixed with 0.2 mL of metaphosphoric acid (250 g/L) and stored at 4 °C for 30 min. The mixture was then centrifuged at 21,000× *g* for 10 min at 20 °C, and the supernatant was injected into a gas chromatograph (HP 6890, Hewlett-Packard Co., Palo Alto, CA, USA) provided with a flame ionization detector and capillary column (Nukol Fused silica capillary column 30 m × 0.25 mm × 0.2 μm, Supelco Inc., Bellefonte, PA, USA). The oven, injector, and detector temperature were set to 90 °C to 180 °C and 210 °C, respectively. Nitrogen was used as the carrier gas at a flow rate of 40 mL/min.

The serum samples were analyzed for total protein, alanine transamilnase aspartate transamilnase, glucose, triglycerides, total cholesterol, non-esterified fatty acids, creatine, blood urea nitrogen, calcium, inorganic phosphate, magnesium, and albumin with kits provided by Wako Pure Chemical Industries, Limited (Osaka, Japan) and a clinical auto-analyzer (Toshiba Accute Biochemical Analyzer-TBA-40FR, Toshiba Medical Instruments, Tokyo, Japan).

### 2.4. Data Processing and Statistical Analysis

All statistical analyses were peformed using the PROC MIXED procedure of SAS (SAS Institute Inc., Cary, NC, USA), as recommended by Seo et al. [19]. Analysis of variance was conducted to detect differences among treatments (i.e., concentrate mixes). To test the effects of different treatments on BW, DMI, and rumen parameters, the data were analyzed as repeated measures to account for the correlation between repeated measurements within each animal. Through this approach, the treatment effects on the monthly averages of BW and DMI and the daily averages of rumen parameters were determined. For this analysis, no specific structure was assumed for the variance–covariance matrix. In addition, the steers’ estimated genomic breeding value for carcass weight was included in the model as a covariate when analyzing growth to adjust for genetic effects on growth rate. Tukey’s multiple range test was used to assess differences between groups. Statistical significance was defined at *p* ≤ 0.05, and a tendency was considered at *p* ≤ 0.1.

## 3. Results

The average daily gain significantly differed by treatment (Table 3). The ADG increased quadratically (*p* = 0.025) as the ME concentration in the concentrate mix increased. Steers in the HEC group showed a 16.5% higher ADG compared with those in the LEC and MEC groups. However, no significant difference was found between the LEC and MEC groups.

The concentrate intake slightly but significantly decreased as the ME concentration in the concentrate mix increased (*p* = 0.030), although no significant difference among the treatment means was observed. Interestingly, a significant quadratic pattern was observed in both forage and total DMI intake (Table 3, *p* < 0.001) due to differences in forage intake. Compared with the LEC and MEC groups, which did not significantly differ, forage DMI was significantly reduced in the HEC group by 49% (*p* < 0.001). Consequently, total DMI was also reduced by 12% in the HEC group compared with the LEC and MEC groups (*p* < 0.001). A similar pattern was observed in the feed conversion ratio (FCR), where a 28% reduction in FCR—a 28% increase in feed efficiency—was observed in the HEC group compared with the LEC and MEC groups (*p* = 0.001).

A notable difference in fiber digestibility was observed among the energy treatments (Table 4). Both aNDF and ADF digestibilities linearly increased with higher energy levels in the concentrate mix (*p* = 0.008 and 0.032, respectively). Specifically, NDF digestibility was significantly higher in the HEC group compared to the LEC group, with an increase of 7.1% p. The digestibility of organic matter (*p* = 0.077) and CP (*p* = 0.051) also tended to improve with higher energy levels. No significant differences were observed in the digestibility of DM and EE across the treatments.

Significant differences in ruminal VFA concentrations were observed among the treatments (Table 5). Similar to the pattern seen in intakes, a significant quadratic response—an increase followed by a decrease—was observed in the total VFA concentration and the acetate proportion as the energy concentration in the concentrate mix increased (*p* < 0.05). The total VFA concentration was 12.4 mM (18.7%) lower in the HEC group than in the MEC group; however, no significant differences were found between the LEC and HEC groups or between the LEC and MEC groups. Similarly, the proportion of acetate was 22 mol/mol lower in the HEC group compared with the MEC group (Table 5). On the contrary, the proportion of propionate was 22 mol/mol higher in the HEC group compared with the LEC and MEC groups (*p* < 0.001). Consequently, the acetate–propionate ratio was 13% reduced in the HEC group compared with the LEC and MEC groups (*p* < 0.001). The butyrate concentration also decreased linearly as the energy content in the concentrate mix increased (*p* = 0.006).

The effects of the energy level on blood metabolites are presented in Table 6. Creatinine levels increased linearly (*p* = 0.009) with higher energy levels, with the steers fed the HEC diet showing a creatinine concentration that was 0.2 mg/dL higher than that in the LEC group. Although not statistically significant, blood glucose, non-esterified fatty acid, and albumin concentrations tended to increase as the dietary energy levels rose (*p* < 0.1).

## 4. Discussion

Given energy’s role in growing and fattening, supplying an optimal energy level without compromising rumen health is essential for Hanwoo beef cattle [2]. However, results from previous studies on providing Hanwoo steers with varying energy levels are inconsistent. This study, therefore, aimed to explore the effects of dietary energy levels in concentrate mixes on comprehensive physiological parameters in Hanwoo steers.

We observed significantly higher ADG and lower forage and total DMI with the highest-energy-containing concentrate mix (HEC). Consequently, the HEC group showed a significantly higher feed efficiency than the other groups. This result aligns with Chen et al. [11], who demonstrated a positive response in growth rate with increased dietary energy levels. Reduced forage intake has often been observed in previous studies [25,26,27,28], consistent with the theory that high-energy diets limit DMI through a metabolic feedback mechanism. This mechanism suggests that as the energy density of the diet increases, DMI decreases to avoid excessive total energy intake [29].

The higher blood creatinine level supports the increased ADG in the HEC group. Creatinine is a product of creatine metabolism in muscle, where creatine phosphate is converted in to creatinine [30]. The blood creatinine concentration is known to correlate with muscle mass [31]. Our result aligns with Sadeghi et al. [32], who found that heifers fed a high-energy diet had higher creatinine levels compared to those fed a low-energy diet. Similarly, Kim et al. [33] reported increased creatinine levels as Hereford heifers gained weight. Lawrence et al. [34] also observed higher creatinine levels in efficient (low residual feed intake) heifer groups, indicating that efficient animals may exhibit higher creatinine levels, as seen in the current study.

Significantly higher fiber digestibility and trends of higher CP and OM digestibilities suggest that the HEC group was superior in extracting available nutrients from the diet. The HEC group had a reduced overall feed intake, which could subsequently improve nutrient digestibility due to a longer retention time and more efficient fermentation in the rumen [35]. Fiber digestibility is enhanced by an increased retention time of the fiber in the rumen. Ruminal retention time of feed particles is primarily determined by feed intake [36]. A reduction in feed intake may decrease the passage rate of feed particles out of the rumen, leading to improved fiber digestibility. Additionally, reduced forage intake in the HEC group lowered the intake of forage NDF, shifting the fiber source to a higher proportion of concentrate NDF, which is typically more digestible than forage NDF due to its lower lignin content and simpler structure [37].

The mean ruminal pH did not differ among the treatments, while the total VFA concentration and molar proportion of acetate, propionate, and, thus, the acetate–propionate ratio significantly differed in the HEC group compared to the LEC and MEC groups, likely due to lower forage and total DMI in the HEC group. The proportion and molar concentrations of VFA depend on the feed type and nutrient levels in the diet [38,39,40]. The total VFA concentration was lower in the HEC group, which may primarily be attributed to the lower carbohydrate consumption in this group compared to the others. The HEC group also showed a lower acetate concentration alongside a higher propionate concentration in the rumen, leading to a lowered acetate–propionate ratio, likely due to reduced forage consumption [41].

Moreover, butyrate and valerate differed significantly among the treatment groups. The butyrate concentration was lower in the HEC group compared to the LEC group, which may be attributed to differences in starch intake between the two groups, as butyr-ate levels are positively correlated with starch supply [42]. The molar concentration of valerate was higher in the HEC group compared to the MEC group, possibly due to differences in nutrient composition [43]. A low fiber-to-starch ratio can enhance valerate production along with other VFAs [44]. Although the MEC treatment contained more starch and fiber, the lower fiber content in the HEC diet may have contributed to increased valeric acid production.

Unexpectedly, the MEC group exhibited similar ADG and feed-to-gain ratios as the LEC group. One possible explanation is the higher starch concentration in the LEC diet. The amount of starch intake is closely related with growth rate [11]. More importantly, the ambient temperature during this trial was extremely high, with nighttime temperatures exceeding 25 °C for a month. Substituting dietary fiber with starch and fat can lower heat production and increase energy efficiency in ruminants [45,46]. The efficiency of energy utilization is reduced during heat stress [45], resulting in an overall reduced ADG in the present feeding trial.

## 5. Conclusions

In conclusion, feeding Hanwoo steers a high-energy concentrate mix (up to 11.0 MJ/kg DM) during the early fattening period improved fiber digestibility, growth rate, and feed efficiency without adverse effects. These findings suggest potential to shorten the fattening period and reduce production costs, though further studies with cost–benefit analyses are needed. Additionally, long-term research should explore gut microbiota shifts and carcass composition changes to better understand the impact of high-energy diets.

## Figures and Tables

**Table 1:** Diet formulation of the experimental concentrate mixes.

Ingredients (g/kg DM) ^1^	Treatment ^2^		
	LEC	MEC	HEC
Corn, ground	123	17	26
Wheat, ground	81	121	108
Dehulled brown rice	19	0	19
Lupin seed	31	0	51
Soybean meal	132	221	93
Rapeseed meal	50	30	30
Copra meal	102	20	50
Palm kernel meal	64	84	105
Corn germ meal	49	53	52
Wheat brewers grains	0	0	20
DDGS	20	0	13
Vegetable oil	17	22	0
Animal fat	0	4	16
Soy hull	102	200	87
Corn gluten feed	0	0	235
Cottonseed hull	136	104	0
Beet pulp	0	51	0
Wheat bran	0	0	29
Molasses	20	36	32
CMS	25	11	11
Limestone	16	11	17
Salt	2	2	3
Sodium bicarbonate	5	13	0
Calcium carbonate	3	0	0
Vitamin and mineral mix ^3^	3	2	3

^1^ DDGS: distiller’s dried grains with soluble, CMS: condensed molasses soluble. ^2^ LEC: low-energy concentrate mix, MEC: medium-energy concentrate mix, HEC: high-energy concentrate mix. ^3^ 33,330,000 IU/kg vitamin A, 40,000,000 IU/kg vitamin D, 20.86 IU/kg vitamin E, 20 mg/kg Cu, 90 mg/kg Mn, 100 mg/kg Zn, 250 mg/kg Fe, 0.4 mg/kg I, and 0.4 mg/kg Se.

**Table 2:** Analyzed chemical composition (g/kg DM or as stated) of the experimental diets.

Items ^1^	Concentrate Mix ^2^			Tall Fescue
	LEC	MEC	HEC	
DM, g/kg as fed	886	891	898	898
OM	932	921	928	936
CP	168	205	213	66
SOLP	66	63	96	25
NDICP	37	35	39	15
ADICP	25	17	20	9
Crude fiber	155	156	112	364
aNDF	351	362	346	631
ADF	206	199	182	418
ADL	60	46	51	60
Starch	264	182	162	1
Ether extract	41	45	57	11
Ash	68	79	72	64
Ca	8	8	9	2
P	4	8	7	1
Mg	3	3	3	1
K	14	12	13	19
S	3	3	4	1
Na	5	2	2	1
Cl	6	5	5	3
TDN	685	698	719	556
ME, MJ/kg DM	10.4	10.8	11.0	8.3
NEm, MJ/kg DM	6.9	7.2	7.5	4.7
NEg, MJ/kg DM	4.3	4.6	4.8	2.4
Total carbohydrates	724	671	658	859
NFC	409	344	351	242
Carbohydrate fraction, g/kg carbohydrate				
CA	185	191	194	148
CB1	365	271	246	1
CB2	15	51	93	133
CB3	235	324	279	551
CC	200	163	187	167
Protein fraction, g/kg CP				
PA + B1	393	307	451	379
PB2	388	520	367	402
PB3	71	89	87	77
PC	148	84	95	142

^1^ DM: dry matter, OM: organic matter, CP: crude protein, SOLP: soluble CP, NDICP: neutral-detergent-insoluble CP, ADICP: acid-detergent insoluble CP, aNDF: neutral detergent fiber analyzed using a heat-stable amylase and expressed inclusive of residual ash, ADF: acid detergent fiber, ADL: acid detergent lignin, TDN: total digestible nutrients, ME: metabolizable energy, NEm: net energy for maintenance, NEg: net energy for growth, NFC: non-fiber carbohydrate, CA: carbohydrate A fraction; water-soluble carbohydrates, CB1: carbohydrate B1 fraction; starch, CB2: carbohydrate B2 fraction; soluble fiber, CB3: carbohydrate B3 fraction; available insoluble fiber, CC: carbohydrate C fraction; unavailable carbohydrate, PA + B1: protein A and B1 fractions; soluble CP, PB2: protein B2 fraction; intermediate degradable CP, PB3: protein B3 fraction; slowly degradable fiber-bound CP, PC: protein C fraction; unavailable CP. ^2^ LEC: low-energy concentrate mix, MEC: medium-energy concentrate mix, HEC: high-energy concentrate mix.

**Table 3:** Effects of energy level in concentrate mix on growth performance in Hanwoo steers.

Items ^1^	Treatment ^2^			SEM	*p*-Value		
	LEC	MEC	HEC		Mean	Linear	Quadratic
Initial BW, kg	500	502	500	13.8	0.996	0.996	0.935
Final BW, kg	568	570	579	15.2	0.850	0.599	0.655
ADG, g/d	696.0 ^b^	692.6 ^b^	809.0 ^a^	34.11	0.035	0.023	0.025
DMI, kg/d							
Concentrate mix	8.03	8.01	7.71	0.134	0.060	0.030	0.050
Forage	1.80 ^a^	2.19 ^a^	1.08 ^b^	0.141	<0.001	<0.001	<0.001
Total	9.84 ^a^	10.20 ^a^	8.79 ^b^	0.159	<0.001	<0.001	<0.001
FCR	14.48 ^a^	15.53 ^a^	10.84 ^b^	0.806	0.001	0.003	<0.001

^1^ BW, body weight; ADG, average daily gain; DMI, dry matter intake; FCR, feed conversion ratio, DMI (g)/ADG (g). ^2^ LEC: low-energy concentrate mix, MEC: medium-energy concentrate mix, HEC: high-energy concentrate mix. ^a,b^ Means that do not have common superscripts differed significantly within the treatments (*p* < 0.05).

**Table 4:** Effects of energy level in concentrate mix on nutrient digestibility in Hanwoo steers.

Items ^1^	Treatment ^2^			SEM	*p*-Value		
	LEC	MEC	HEC		Mean	Linear	Quadratic
DM, %	73.75	75.06	76.92	1.543	0.375	0.172	0.410
OM, %	72.43	74.00	76.81	1.603	0.190	0.077	0.239
CP, %	76.51	77.85	81.93	1.771	0.120	0.051	0.130
EE, %	80.94	89.16	86.67	4.203	0.395	0.354	0.683
aNDF, %	60.45 ^b^	61.70 ^ab^	67.54 ^a^	1.587	0.018	0.008	0.023
ADF, %	54.57	59.53	60.04	1.593	0.060	0.032	0.824

^1^ DM: dry matter, OM: organic matter, CP: crude protein, EE: ether extract, aNDF: neutral detergent fiber analyzed using a heat-stable amylase and expressed inclusive of residual ash, ADF: acid detergent fiber. ^2^ LEC: low-energy concentrate mix, MEC: medium-energy concentrate mix, HEC: high-energy concentrate mix. ^a,b^ Means that do not have common superscripts significantly differed within the treatments (*p* < 0.05).

**Table 5:** Effects of energy level in concentrate mix on rumen characteristics in Hanwoo steers.

Items ^1^	Treatment ^2^			SEM	*p*-Value		
	LEC	MEC	HEC		Mean	Linear	Quadratic
pH	6.60	6.59	6.52	0.056	0.505	0.287	0.345
NH_3_-N, mg/dL	13.88	14.11	14.01	1.237	0.990	0.932	0.953
Total VFA, mM	72.32 ^ab^	78.57 ^a^	66.17 ^b^	2.978	0.016	0.148	0.004
Molar proportions, mmol/mol							
Acetate	641.0 ^ab^	647.2 ^a^	624.6 ^b^	7.89	0.019	0.036	0.008
Propionate	165.9 ^b^	169.3 ^b^	190.1 ^a^	4.47	<0.001	<0.001	<0.001
Isobutyrate	13.1	12.9	14.2	0.99	0.611	0.435	0.359
Butyrate	151.1 ^a^	143.6 ^ab^	139.4 ^b^	4.21	0.019	0.006	0.341
Isovalerate	16.1	15.1	17.5	1.20	0.386	0.413	0.172
Valerate	14.1 ^ab^	12.5 ^b^	15.7 ^a^	1.07	0.039	0.185	0.011
Acetate/Propionate	3.87 ^a^	3.83 ^a^	3.34 ^b^	0.118	<0.001	<0.001	<0.001

^1^ NH_3_-N, ammonia; VFA, volatile fatty acid. ^2^ LEC: low-energy concentrate mix, MEC: medium-energy concentrate mix, HEC: high-energy concentrate mix. ^a,b^ Means that do not have common superscripts differed significantly within the treatments (*p* < 0.05).

**Table 6:** Effects of energy level in concentrate mix on blood metabolites in Hanwoo steers.

Items ^1^	Treatment ^2^			SEM	*p*-Value		
	LEC	MEC	HEC		Mean	Linear	Quadratic
Total protein, g/dL	5.8	5.9	5.9	0.12	0.800	0.517	0.836
Urea, mg/dL	16.9	17.3	17.3	0.66	0.871	0.673	0.958
Glucose, mg/dL	58.1	58.6	61.5	1.21	0.115	0.054	0.102
NEFA, mEq/L	0.12	0.15	0.16	0.013	0.156	0.064	0.607
Albumin, mg/dL	2.9	2.9	3.0	0.04	0.122	0.083	0.070
Creatinine, mg/dL	1.0 ^b^	1.1 ^ab^	1.2 ^a^	0.04	0.019	0.009	0.651
Triglyceride, mg/dL	14.2	14.7	13.6	1.13	0.773	0.687	0.478
GOT, U/L	54.4	50.7	52.2	2.57	0.588	0.540	0.683
GPT, U/L	17.9	17.8	17.3	0.69	0.815	0.542	0.647
Cholesterol, mg/dL	112.5	111.6	107.0	6.11	0.794	0.530	0.599
Calcium, mg/dL	8.1	8.2	8.1	0.06	0.386	0.913	0.216
Phosphorus, mg/dL	6.2	6.2	6.1	0.16	0.794	0.568	0.553
Magnesium, mg/dL	2.0	1.9	2.0	0.05	0.745	0.823	0.459

^1^ NEFA: non-esterified fatty acid, GOT: glutamic oxaloacetic transaminase, GPT: glutamic pyruvic transaminase. ^2^ LEC: low-energy concentrate mix, MEC: medium-energy concentrate mix, HEC: high-energy concentrate mix. ^a,b^ Means that do not have common superscripts significantly differed within the treatments (*p* < 0.05).

## Data Availability

Upon request, the data used in this study can be provided by the corresponding author.
